# Effect of post-extubation high-flow nasal cannula combined with respiratory training versus conventional oxygen therapy on postoperative pulmonary complications in patients after major abdominal surgery: protocol for a single-centre randomized controlled trial

**DOI:** 10.1186/s13063-023-07311-2

**Published:** 2023-06-13

**Authors:** Biao Jin, Mengjing Yao, Wenjuan Shen, Le Fu, Ping Liu, Xu Zheng, Tiexiang Zhan, Liang Luo

**Affiliations:** Department of Intensive Care, The Seventh Affiliated Hospital, Sun Yet-Sen University, ShenZhen, China

**Keywords:** Postoperative pulmonary complications, High-flow nasal cannula, Conventional oxygen therapy, Randomized controlled trial, Major upper abdominal surgery

## Abstract

**Background:**

Nearly 234 million patients undergo surgery each year, and 1.3 million among them develop complications. Patients undergoing major upper abdominal surgery (operation time > 2 h) have a really high incidence of postoperative pulmonary complications (PPCs). The occurrence of PPCs seriously affects the outcomes of patients. High-flow nasal cannula (HFNC) is as effective as noninvasive ventilation (NIV) in preventing postoperative hypoxaemia and respiratory failure. Respiratory training using positive expiratory pressure (PEP) Acapella (Choice) has been shown to help patients with rapid recovery from postoperative atelectasis. However, no relevant randomized controlled studies have been conducted to clarify the effect of HFNC combined with respiratory training in the prevention of PPCs. This study aims to investigate whether the use of HFNC combined with respiratory training could reduce the incidence of PPCs within 7 days after major upper abdominal surgery compared to that with conventional oxygen therapy (COT).

**Methods:**

This is a randomized controlled single-centre trial. A total of 328 patients who undergo major abdominal surgery will be included. Subjects who fulfil the eligible criteria will be randomly assigned into the combination treatment group (Group A) or COT group (Group B) after extubation. The interventions will begin within 30 min of extubation. Patients in Group A will receive HFNC for at least 48 h and respiratory training three times a day for at least 72 h. Patients in Group B will receive oxygen therapy through a nasal catheter or mask for at least 48 h. Our primary endpoint is the incidence of PPCs within 7 days, and the secondary outcome measures include 28-day mortality, reintubation rate, length of hospital stay, and all-cause mortality within 1 year.

**Discussion:**

This trial would help provide evidence on the effectivity of applying HFNC combined with respiratory training for the prevention of PPCs in patients undergoing major upper abdominal surgery. The objective of this study is to determine the optimal treatment approach to improve the prognosis of patients undergoing surgery.

**Trial registration:**

ChiCTR2100047146. Registered on 8 June 2021. Retrospectively registered.

**Supplementary Information:**

The online version contains supplementary material available at 10.1186/s13063-023-07311-2.

## Administrative information

Note: The numbers in curly brackets in this protocol refer to SPIRIT checklist item numbers. The order of the items has been modified to group similar items (see http://www.equator-network.org/reporting-guidelines/spirit-2013-statement-defining-standard-protocol-items-for-clinical-trials/).

**Table:** 

Title {1}	Effect of post-extubation high-flow nasal cannula combined with respiratory training versus conventional oxygen therapy on postoperative pulmonary complications in patients after major abdominal surgery: protocol for a single-centre randomized controlled trial
Trial registration {2a and 2b}.	ChiCTR2100047146. Registered on 8 June 2021. http://www.chictr.org.cn/index.aspx
Protocol version {3}	Version 1 of 14–04-2021
Funding {4}	The trial is supported by “The Seventh Affiliated Hospital, Sun Yat-sen University Clinical Research 735 Program”.
Author details {5a}	Department of Intensive Care, The Seventh Affiliated Hospital,Sun Yet-sen University, ShenZhen, China.
Name and contact information for the trial sponsor {5b}	Liang Luo, Department of Intensive Care, The Seventh Affiliated Hospital, Sun Yet-sen University, ShenZhen, China. luoliang@mail.sysu.edu.cn
Role of sponsor {5c}	Liang Luo all contributed to the design and management of the study protocol.

## Introduction

### Background and rationale {6a}

Nearly 234 million patients undergo surgery every year, among whom 1.3 million patients experience complications [1]. Postoperative pulmonary complications (PPCs), including hypoxemia, pneumonia, respiratory failure, atelectasis are more common than cardiovascular complications following surgery [2, 3]. Such complications have high incidence ranging from 6 to 80% and are a leading cause of poor surgical outcomes [4]. Studies estimate that nearly one million PPCs occurred annually in the United States, resulting in 46,200 deaths and 480,000 additional hospital days [5]. Despite the intraoperative use of protective ventilation practices, Ana et al. [6] found PPCs more commonly in patients with surgery lasting more than 2 h and an American Society of Anaesthesiologists (ASA) status 3. Most PPCs occurred within 7 days of surgery. Even mild PPCs can significantly affect the postoperative mortality and therefore deserve close attention and intervention in clinical practice.

The incidence of PPCs is up to 40% in patients with an ASA status 3, and thoracic or upper abdominal surgeries [7]. The pathogenesis of PPCS includes a decreased functional residual capacity (FRC), atelectasis, ineffective cough and abnormal respiratory control [8, 9]. Effective prevention of PPCs is strongly associated with a shorter hospital stay and decreased mortality. Postoperative factors including respiratory support, breathing training, and physical therapy increasingly affect the incidence of PPCs. Current respiratory support methods include noninvasive ventilation (NIV), high-flow nasal cannula (HFNC), and conventional oxygen therapy (COT). NIV is effective in preventing and treating respiratory failure and reducing the rate of postoperative tracheal reintubation in different populations [10, 11]. However, recent research shows that the routine prophylactic use of NIV after major abdominal surgery does not reduce the incidence of PPCs [12]. Furthermore, some patients cannot effectively cooperate and tolerate NIV. In addition, the use of NIV after abdominal surgery may increase the risk of gastrointestinal fistulas. Therefore, routine use of prophylactic post-operative NIV is not recommended.

HFNC uses heated humidification, high flow (up to 60L/min), and a high oxygen concentration (up to 100%). At high flow rates, HFNC is believed to increase positive end-expiratory pressure by 4–6 cmH_2_O [13]. Besides patients feel more comfortable using HFNC than with NIV or COT. Studies have shown that HFNC is not inferior to NIV and COT in populations at high risk for PPCs in preventing hypoxaemia and respiratory failure [14]. Frat et al. compared the effects of HFNC, COT, and NIV in hypoxic respiratory failure and found that the 90-day mortality rate was lowest in the HFNC group, although no significant difference was observed in the reintubation rate [15]. Compared with COT, HFNC provided better oxygenation, better comfort, and lower reintubation rate at the same oxygen concentration [16]. Recent study indicates the use of HFNC did not reduce the rate of reintubation in comparison to COT; however, HFNC yielded less frequent use of rescue NIV [17]. HFNC is not inferior to NIV in preventing reintubation and post-extubation respiratory failure in populations at high risk of reintubation [18]. Recent guidelines of the European Respiratory Society suggest using either HFNC or NIV in postoperative patients at high risk of respiratory complications. Additionally, they also suggest using either COT or HFNC in postoperative patients at low risk of respiratory complications [19].

In addition, postoperative diaphragmatic dysfunction is also the pathophysiological basis of PPCs. Preoperative inspiratory training is believed to reduce the occurrence of PPCs after major thoracic and abdominal operations [20]. The Acapella vibrating ventilation therapy produces a positive pressure of 5–15 cmH_2_O at the end of the breath to maintain lung expansion. It can promote sputum excretion, strengthen respiratory muscle, and reduce the incidence of alveolar collapse, pneumonia, and hypoxaemia. Studies have shown that this therapeutic system is as effective as other airway clearance techniques in improving respiratory symptoms, expectoration, lung capacity, and health-related quality of life [21]. However, previous studies have shown that the routine use of incentive spirometry for breathing training after major upper abdominal surgery did not reduce the incidence of PPCs [22, 23]. Well-designed clinical trials are urgently required for further validation.

These two interventions have theoretical benefits for the prevention of PPCs. However, previous clinical studies have shown that neither HFNC nor breathing training after surgery shows a significant difference in the occurrence of PPCs. In our clinical practice, we observed that patients at high risk for PPCs recovered faster using HFNC combined with respiratory training (positive expiratory pressure [PEP] Acapella Choice). Therefore, a pilot trial of 12 cases of the combined use of HFNC and Acapella breathing training was conducted. The incidence rate of PPCs was 50% (3/6) in the COT group, while the incidence of PPCs was 16.7% (1/6) and the diaphragm function recovered faster in the combination treatment group. To date, no randomized controlled trials have verified the role of HFNC combined with respiratory training in preventing PPCs. We believe that this randomized controlled study will provide new evidence for the prevention of PPCs and improve the long-term prognosis.

### Objectives {7}

Primary objective: To reduce the incidence of PPCs within 7 days of surgery compared with routine use of COT and apply these findings to clinical practice.

Secondary objective: To investigate whether HFNC combined with respiratory training can improve postoperative outcomes of patients, such as reducing 28-day mortality, 1-year all-cause mortality, reintubation rates, and length of hospital stay after major upper abdominal surgery.

### Trial design {8}

The study is a parallel, single-blind, randomized controlled clinical trial (RCT). Patients who fulfil the eligibility criteria and signed the informed consent will be randomly assigned to either the combination treatment group (Group A) or the COT group (Group B) in a 1:1 ratio. Periodic evaluation will be made daily within 1 week, at discharge, the 28^th^ day, and 1 year after the surgery. We hypothesize that Group A would have a lower incidence of PPCs compared to Group B.

## Methods: participants, interventions, and outcomes

### Study setting {9}

Patients who undergo elective abdominal surgery (operation time > 2 h) at the Seventh Affiliated Hospital, Sun Yat-sen University, will be recruited and will be considered for inclusion if they meet the criteria as defined below.

### Eligibility criteria {10}

#### Inclusion criteria

1. age ≥ 18 years, (2) surgery time ≥ 2 h, (3) abdominal surgical site, and (4) BMI ≤ 35 kg/m[2] (normal, mild obesity, excluding moderate to severe obesity).

#### Exclusion criteria

1. Endotracheal intubation not successfully removed within 24 h after surgery;
2. Obstructive sleep apnoea syndrome (OSAHS);
3. Patients with haemodynamic instability (defined as the need for a norepinephrine dose > 0.5 ug/ kg/min or dopamine > 10ug / kg/min to maintain mean arterial pressure [MAP] ≥ 65 mmHg);
4. Contraindications for the use of the PEP Acapella (Choice) including: inability to follow verbal instructions, active haemoptysis, oesophageal surgery, untreated pneumothorax, and haemodynamic instability.
5. Significant abnormal preoperative chest radiograph or chest CT affecting the identification of PPCs;
6. Abnormal preoperative chest X-ray or CT scan, which affects the discrimination of PPCs;
7. Preoperative impaired consciousness or mental illness (Glasgow score ≤ 14);
8. Participation in other clinical studies;
9. Pregnant woman; and
10.  Informed consent not obtained.

### Who will take informed consent? {26a}

All patients undergoing upper abdominal surgery were initially assessed for eligibility. For elective surgery with an expected duration over than 2 h, the study group will obtain informed consent prior to surgery. Furthermore, for emergency surgery longer than 2 h, an informed consent will be signed after surgery with authorized surrogates. The specific situation of the trial will be explained in detail after the patient has regained consciousness. The participants can communicate with the study group or doctor for any remaining questions at any time.

### Additional consent provisions for collection and use of participant data and biological specimens {26b}

This trial does not involve collecting biological specimens.

## Interventions

### Explanation for the choice of comparators {6b}

The COT group received oxygen by nasal catheter or mask for at least 48 h after extubation.

### Intervention description {11a}

In both groups, implementation of the intervention will start within 30 min of extubation. Postoperative extubation procedures will be performed according to the ATS/ACPP clinical practice guidelines for mechanical ventilation withdrawal. Pain management will be administered to maintain a VAS score of < 3. Decisions regarding other treatment and monitoring will be conducted based on routine clinical practice.

#### Combination treatment group (Group A)

After extubation, patients will receive HFNC therapy for at least 48 h and respiratory training three times a day for at least 3 days.

#### HFNC therapy

Patients will receive oxygen therapy through HFNC. Initial parameters will be set as follows: flow 50L/min, FiO2 100%, T 37.0℃. Then downgrade FiO_2_ to the lowest value to maintain SPO_2_ above 95%. The oxygen flow rate was at least 50L/min in the first 24 h and can be adjusted to 60L/min as needed. The oxygen flow can be reduced to an acceptable level if patients feel uncomfortable. HFNC therapy will last for at least 48 h, then switch to COT.

#### Respiratory training

Respiratory training will be performed using PEP Acapella (Choice), and instruction will be provided by a well-trained physician or respiratory therapist. The specific operation procedures of routine oxygen therapy and videos of respiratory training have already been produced. The implementation of the intervention will be recorded in the Case Report Form.

#### PEP Acapella (choice)

The operation steps are as follow (Patients in the seated or semi-recumbent position):

1. On first use, turn the frequency adjustment dial to the lowest frequency resistance setting marked “1”;
2. Put the nozzle in the mouth, instruct the patient to relax, and then take a deep breath (total lung capacity is not required);
3. Hold breath for 2 ~ 3 s;
4. Exhale slowly, making sure to keep the mouthpiece sealed when exhaling (the exhale time is about 3–4 times longer than the inhale time, no need to exhale forcefully to the “FRC”);
5. Adjust the dial to increase the resistance (adjust clockwise to increase the resistance of the vibrating hole, allowing the patient to exhale at a lower flow rate);
6. Determine the scope;
7. Repeat 10–20 PEP breaths, followed by 2–3 “puff” coughs as needed to cough up secretions; and
8. Repeat the above steps 4 ~ 8 times, no more than 20 min.

Patients can be considered to transfer out of ICU once FiO2 ≤ 35% with SPO_2_ maintained at ≥ 95%.

#### COT group (Group B)

COT would be continued for at least 48 h after extubation and may be continued or stopped after 48 h. Oxygen is transferred through a nasal catheter or a mask (with or without an oxygen storage bag or Venturi system). The oxygen flow is set to maintain SPO_2_ ≥ 95%. Patients can be considered for transfer out of ICU once oxygen flow is < 6L/min with SPO_2_ maintained at ≥ 95%.

### Criteria for discontinuing or modifying allocated interventions {11b}

Participants can withdraw at any time during the trial. And study investigators can decide whether a participant discontinues the trial out of safety concerns, including the following: (1) serious complications such as consciousness disorders; (2) the occurrence of other diseases that affect the efficacy of this trial; and (3) investigators consider the subject unsuitable to continue the study intervention (PEP Acapella).

Other reasons such as death and loss to follow-up may also be considered to terminate the trial. Any modifications of the protocol will be reported to the Ethics Committee and discussed by the study group prior to the implementation.

### Strategies to improve adherence to interventions {11c}

All nurses and physicians involved in this study will be well trained on the specific operation process. Study investigators will visit the participants each day during hospitalization to provide advice on their treatment. Moreover, counsels will also be given on their scheduled clinic visits. Biweekly telephone calls and follow-up at 28^th^ and 1 year after discharge will also be made to improve adherence.

### Relevant concomitant care permitted or prohibited during the trial {11d}

Drugs such as anodyne and expectorant do not affect the trial and can be used according to the guidelines.

### Provisions for post-trial care {30}

Participants will have access to study clinics for post-trial care through the routine health system.

### Outcomes {12}

#### Primary outcome

The primary outcome is the incidence of PPCs within 7 days after surgery. This includes respiratory infections, bronchospasm, aspiration lung injury, pneumonia, atelectasis, pleural exudation, pneumothorax, and acute respiratory failure, according to the EPCO definitions [24]. Each diagnostic criterion is available in the Additional file.

#### Secondary outcome

Secondary outcomes include the following: incidence of PPCs during hospitalization, length of hospital stay, 28-day mortality, NIV or reintubation rates, incidence of surgical complications (surgical site bleeding, infection, wound infection, anastomotic leakage, and surgical reintervention), and all-cause mortality within 1 year.

### Participant timeline {13}

The participant timeline is presented in Table 1.

**Table 1:** Flowchart of patient follow-up

			Primary study period		Follow-up		
Timepoint	Screening D − 7 to D − 1	Inclusion D − 1 to D0	D1 to D7 after randomization	End of hospitaliztion	D28 after randomization	D90 after randomization	1Y after randomization
**Enrollments**	×						
Eligibility screen	×						
Patients information	×						
Informed consent	×						
Randomization		×					
**Interventions**							
HFNC		×	×				
Respiratory training		×	×				
COT		×	×				
**Assessments**							
Baseline variables		×					
Laboratory tests Ultrasound, chest radiography		×	×				
Primary outcomes			×				
Secondary outcomes			×	×			
Complication/adverse events			×	×			
Alive or dead status				×	×	×	×

### Sample size {14}

Based on our previous clinical data (the previously mentioned pilot study) and the latest sample size analysis in the literature (nearly 40% incidence of PPCs), we predict that the incidence of PPCs will be approximately 15% lower in Group A than in Group B [7, 18, 25]. PASS.11 software will be used to compute the sample size. Assuming an α value of 0.025, a *β* value of 0.1, with a maximum tolerated patient loss rate of 10%, 164 patients were required per study group.

### Recruitment {15}

At least 328 patients will be recruited from the Seventh Affiliated Hospital of Sun Yat-sen University through the publication and distribution of text messages, phone calls, and advertisements containing information of the study. Physicians screen potential patients for inclusion and exclusion criteria and register information in the subject registration form. One study investigator checked patients' eligibility for inclusion before randomization.

## Assignment of interventions: allocation

### Sequence generation {16a}

A random number table will be generated using SAS software and assigned according to a random number in a 1:1 ratio by an appointed investigator.

### Concealment mechanism {16b}

The random grouping results (Group A or Group B) corresponding to the enrolment number of the participants will be placed inside the opaque, sealed envelopes.

### Implementation {16c}

**Fig. 1:**
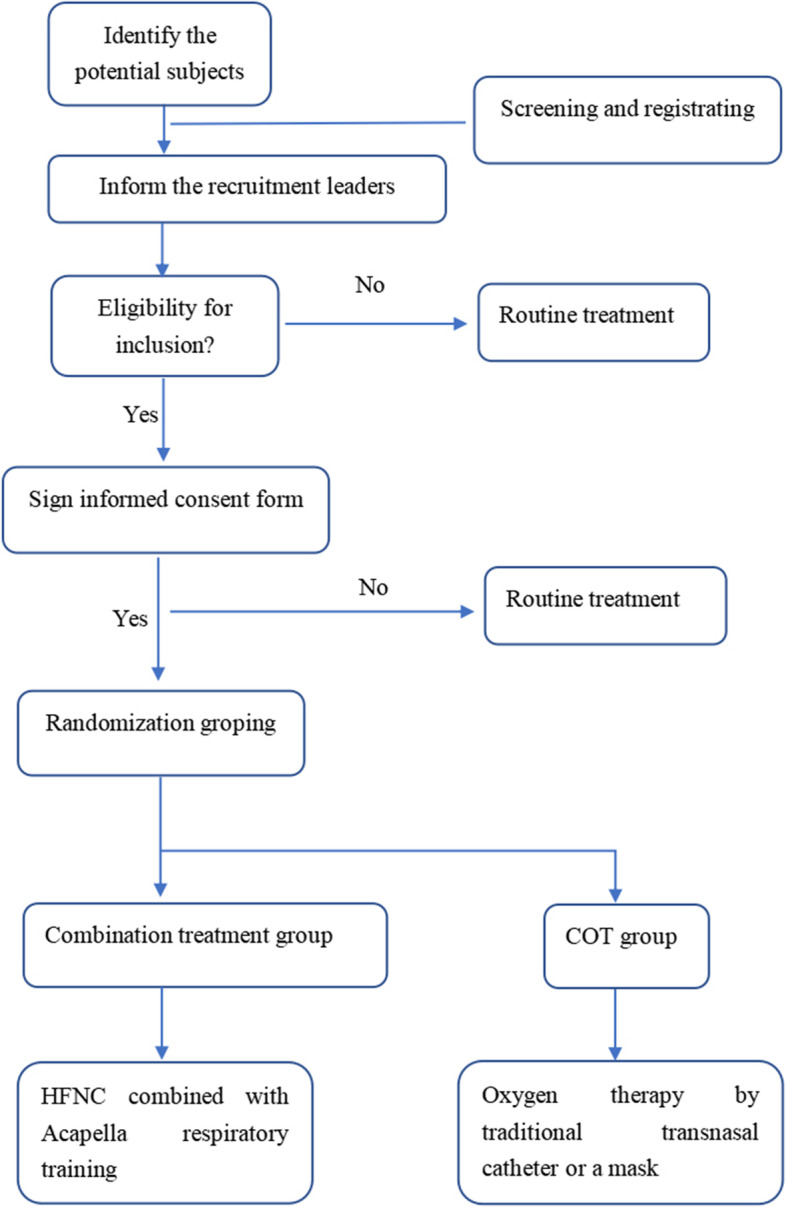
Flowchart of the study

After the eligibility of the participants is confirmed and informed consent is obtained, a trial number will be assigned only after all inclusion and exclusion criteria have been met. Participants will be randomly assigned to the trial (see flowchart in Fig. 1).

## Assignment of interventions: blinding

### Who will be blinded {17a}

Given the nature of the intervention, it is not possible to blind the physicians, nurses, and patients. One critical care physician who is not aware of the clinical research and intervention will collect data on clinical and vital signs. In addition, a team of experts consisting of one ultrasonographer, one radiologist, and one intensivist, have been established to interpret the ultrasound and chest radiograph results. Each member of the team of experts is at least an attending physician and unaware of the patient information.

### Procedure for unblinding if needed {17b}

Unblinding will only be allowed under emergencies such as the incidence of serious adverse effects.

## Data collection and management

### Plans for assessment and collection of outcomes {18a}

Baseline variables such as age, sex, body mass index, and chronic comorbidities will be collected on admission by physicians. Data related to surgical conditions like planned surgical procedure, ARISCAT score, mechanical ventilation will be acquired from the electronic medical record. Laboratory tests will be conducted at the laboratory. Chest X-ray and ultrasound examination will be performed by a radiologist and an ultrasonographer, respectively. All relevant data will be stored in the research folder, accessible only to the study group.

### Plans to promote participant retention and complete follow-up {18b}

At each postoperative visit and breathing guide, the study staff will answer participants’ questions, and recommend measures to promote their recovery. Biweekly telephone calls will be made to check responses for completion of their follow-up.

### Data management {19}

All data will be recorded in the paper case report form (CRF), and.subsequently entered into an electronic database. Data are managed by a designated investigator. Any study folder reflecting personal information of the subjects will be stored in a locked room.

### Confidentiality {27}

The research records and data are identified by the unique code corresponding to names and other personal information of the subjects. Relevant study investigators are only permitted to get access to these information. Data entry into databases, public exchanges, and publication of research results will use numbers instead of names and other personal information.

### Plans for collection, laboratory evaluation, and storage of biological specimens for genetic or molecular analysis in this trial/future use {33}

There will be no biological specimens collected.

## Statistical methods

### Statistical methods for primary and secondary outcomes {20a}

The Shapiro–Wilk test will be used to test whether continuous variables are normally distributed. Normal distribution will be expressed as mean ± standard deviation, and the independent samples t-test will be used to compare between groups. Non-normally distributed continuous variables will be expressed as the median (or interquartile range) using the Mann–Whitney *U* test comparison. Enumeration data will be expressed as the number of cases and percentages, and comparisons between groups will be performed using the chi-squared test. Survival time will be analysed using Kaplan–Meier survival analysis, and comparisons between groups will be performed using the log-rank test. SPSS version 22.0 software (IBM Corp., Armonk, NY, USA) was used for all analyses.

### Interim analyses {21b}

There are no interim analyses planned.

### Methods for additional analyses (e.g., subgroup analyses) {20b}

The treatment effect will be analysed according to the following pre-specified subgroups: (1) open surgery and laparoscopic surgery and (2) patients at high risk for PPCs (ARISCAT score ≥ 45).

### Methods in analysis to handle protocol non-adherence and any statistical methods to handle missing data {20c}

Final analysis will include all participants who had > 75% adherence to the protocol. Participants may withdraw from the trial at any time, then patient data collected up to that moment will be included in the analysis. Multiple imputation will be used to handle missing data if needed.

### Plans to give access to the full protocol, participant level-data and statistical code {31c}

The corresponding author will provide the full protocol, participant level-data and statistical code on appropriate justification complying with national regulations.

## Oversight and monitoring

### Composition of the coordinating centre and trial steering committee {5d}

This is a single-centre research conducted in the Seventh Affiliated Hospital, Sun Yat-sen University. The trial will be coordinated by a team of experts consisting of one ultrasonographer, one radiologist, and one surgeon. Two statisticians will help with the study design and statistical analysis. The study team meets monthly. There is no trial steering committee or stakeholder and public involvement group.

### Composition of the data monitoring committee, its role and reporting structure {21a}

A data security monitoring board (DSMB) will meet monthly to monitor all serious adverse events (SAEs). The DSMB consists of one ultrasonographer, one radiologist, one surgeon, and two statisticians mentioned before. It is independent from the sponsor and will also assess the need for unblinding and termination of the trial for safety consideration.

### Adverse event reporting and harm {22}

The investigators will record all adverse events (AEs) experienced by the trial subjects and immediately report them to the research team, the DSMB and the Ethics Committee. SAEs would be documented in detail with the symptoms, severity, relevance to intervention, time of onset, time of treatment, measures taken, time and manner of follow-up, and outcome, in the SAE report form. If the investigator considers an SAE unrelated to the intervention but potentially related to the study condition (e.g., termination of original treatment or comorbidities in the course of the trial), this relationship would be detailed in the narrative section of the SAE report form. If the intensity of an ongoing SAE or its relationship to the trial intervention changes, a follow-up report would be submitted immediately. If the investigator considers the previously reported SAE misreported, it would be corrected, withdrawn or downgraded in the follow-up report, and reported according to the SAE reporting procedure. AE/SAE would be followed up until the event is appropriately managed, resolved to the baseline level or under grade 1, reach a stable status, or till the outcome (e.g., loss to follow-up, death) is determined. The best possible outcome would be obtained, and its severity and causality (correlation) with reference to the protocol would be assessed.

SAEs of special concern include the following: postoperative acute respiratory failure, postoperative surgical complications (such as incision infection, postoperative bleeding, and anastomotic fistula), complications of Acapella usage (including pneumothorax and respiratory muscle fatigue).

### Frequency and plans for auditing trial conduct {23}

A designated inspector will systematically review the trial-related activities and documentation according to the Medical Research Involving Human Subjects Act (WMO)/Good Clinical Practice (GCP) standards. Annual checks for accuracy and data completion, and standardization of clinical activities will be planned, to assess whether the trials are being conducted in accordance with the protocol and standard operating procedure. Quality control will be performed at each stage of the data processing.

### Plans for communicating important protocol amendments to relevant parties (e.g., trial participants, ethical committees) {25}

Any substantial protocol amendments will be submitted to the ethical committees for approval prior to implementation. Participants will be informed about the changes, to maintain their interests and provision of additional consent if necessary.

### Dissemination plans {31a}

The results of this research will be disseminated in its entirety in international peer-reviewed journals. Both positive and negative results will be reported.

## Discussion

Our study aims to elucidate whether HFNC combined with respiratory training would reduce the incidence of PPCs compared to COT in patients undergoing major upper abdominal surgery. It will also determine whether this will lead to shorter hospital stays and lower medical costs. In addition, the patients in this trial may benefit from increased attention to intensive care measures throughout the entire treatment period. The results of this trial would be different from those of previous research cited above. This is because, so far, there has been no RCT on HFNC combined with respiratory training in postoperative patients. The OPERA trial [26], one of the largest trials investigating the potential role of HFNC in preventing postoperative hypoxaemia, randomized 220 patients after abdominal surgery to receive HFNC or standard oxygen therapy. No significant difference was found between the two groups in terms of absolute reduction in hypoxaemia risk. However, the duration of HFNC treatment in this study was 15 h (12–18 h), while postoperative respiratory failure mostly occurred within 72 h after surgery. Furthermore, the lowest FRC value is usually observed at 1–2 days after upper abdominal surgery, then slowly return to normal after 5–7 days. As mentioned above, decreased FRC and atelectasis are the main pathogenesis of PPCs. Therefore the duration of HFNC in this trial is short, and may be insufficient to show the efficacy on PPC prevention. In our study, patients in Group A will receive HFNC for at least 48 h and respiratory training, three times a day for at least 3 days, which may effectively prevent the occurrence of PPCs.

There are potential risks in this trial. On the one hand, patients at high risk of PPCs may be randomly assigned to the COT Group, which may lead to the aggravation of the disease. However, there are no optimal choice of postoperative oxygen therapy at present. Either HFNC or COT is a routine treatment approach and is unlikely to cause a significant adverse effect on the patient's condition. This study will also involve the timely observation and treatment of these patients. On the other hand, the use of Acapella PEP (choice) treatment system may result in the aggravation of respiratory symptoms in some patients, a respiratory therapist will guide them on how to perform breathing exercises, based on their specific situations.

In conclusion, we hope that this trial will validate the preferred efficacy of HFNC combined with respiratory training compared to COT in reducing the incidence of PPCs and improving clinical outcomes in patients undergoing major abdominal surgery. This may help to provide favourable evidence for the inclusion of application of HFNC and respiratory training in clinical guidelines.

## Trial status

The trial was registered on 8 June 2021 as ChiCTR2100047146. (http://www.chictr.org.cn/index.aspx) The first patient was randomized on 1 November 2021 and enrolment is expected to be completed in August 2025.

## Supplementary Information

**Additional file 1:** **Table S1.** Definitions of postoperative pulmonary complications

## Data Availability

The datasets used and analysed during the current study are available from the corresponding author on reasonable request. The corresponding author will provide the full protocol, participant level-data and statistical code on appropriate justification complying with national regulations.(31c)

## Abbreviations

PPCs: Postoperative pulmonary complications
HFNC: High-flow nasal cannula
NIV: Noninvasive ventilation
COT: Conventional oxygen therapy
PEP: Positive expiratory pressure
RCT: Randomized controlled trial
EPCO: European Perioperative Clinical Outcome
ASA: American Society of Anaesthesiologists
MAP: Mean arterial pressure
ARISCAT: Assess Respiratory Risk in Surgical Patients in Catalonia
AE: Adverse event
SAE: Serious adverse event
DSMB: Data security monitoring board
