# Supplementary material for: Effect of post-extubation high-flow nasal cannula combined with respiratory training versus conventional oxygen therapy on postoperative pulmonary complications in patients after major abdominal surgery: protocol for a single-centre randomized controlled trial
Source: Trials. 2023 Jun 13;24:396. doi: 10.1186/s13063-023-07311-2 (PMC10262414; doi:10.1186/s13063-023-07311-2)
Supplement: Supplementary file 1 — Additional file 1: Table S1. Definitions of postoperative pulmonary complications [file 13063_2023_7311_MOESM1_ESM.docx]

Table S1. Summary of primary outcome indicators.

| **Primary outcome** | **Definitions** |
| --- | --- |
| Respiratory infections | Patient has received antibiotics for a suspected respiratory infection and met one or more of the following criteria: new or changed sputum, new or changed lung opacities, fever, WBC> 12×10^9^/L. |
| Bronchospasm | Newly detected expiratory wheezing treated with bronchodilators. |
| Aspiration pneumonitis | Acute lung injury after the inhalation of regurgitated gastric contents. |
| Atelectasis | Lung opacification with a shift of the mediastinum, hilum or hemidiaphragm toward the affected area, and compensatory over-inflation in the adjacent non-atelectatic lung. |
| Pleural effusion | Chest CXR shows dulling of the costophrenic angle, loss of sharp contours on the ipsilateral side when upright, displacement of adjacent anatomical structures, or (supine) blurring of one side of the hemithorax with preserved vascular shadows. |
| Pneumothorax | Air in the pleural space with no vascular bed surrounding the visceral pleura. |
| Acute respiratory failure | At least one of the items:   1. PaO_2_＜60mmHg under air inhalation or PaO_2_/FiO_2_＜300mmHg, SPO_2_＜90% under oxygen inhalation combined at least one of the following:   Severe dyspnea, respiratory distress; auxiliary respiratory muscles involved in respiration; paradoxical movements of chest and abdomen; respiratory rate > 25 beats/min; respiratory acidosis PH < 7.30; PaCO2 > 50mmHg;   1. Reintubation or non-invasive ventilator-assisted ventilation (NIV) within 7 days due to respiratory distress, hypoxemia, hypercapnia, respiratory acidosis. |
